# Screening for Causative Mutations of Major Prolificacy Genes in Iranian Fat-Tailed Sheep

**DOI:** 10.22074/ijfs.2018.5247

**Published:** 2018-01-15

**Authors:** Ramin Abdoli, Seyed Ziaeddin Mirhoseini, Navid Ghavi Hossein-Zadeh, Pouya Zamani

**Affiliations:** 1 Department of Animal Science, Faculty of Agricultural Sciences, University of Guilan, Rasht, Iran; 2 Department of Animal Science, Faculty of Agriculture, Bu-Ali Sina University, Hamedan, Iran

**Keywords:** Fertility, Litter Size, Single Nucleotide Polymorphism, Sheep

## Abstract

**Background:**

The presence of different missense mutations in sheep breeds have shown that the bone morphogenetic protein receptor 1B (BMPR1B), bone morphogenetic protein 15 (BMP15) and growth differentiation factor 9 (GDF9) genes play a vital role in ovulation rate and prolificacy in ewes. Therefore, the present study aims to investigate BM- PR1B, BMP15 and GDF9 gene mutations in prolific ewes of Iranian fat-tailed Lori-Bakhtiari sheep.

**Materials and Methods:**

In the present experimental study, genomic DNA was extracted from whole blood of 10 prolific Lori-Bakhtiari ewes with at least two twinning records in the first four parities to identify point mutations of the BMPR1B, BMP15 and GDF9 genes, using DNA sequencing.

**Results:**

The results obtained from DNA sequencing showed a new synonymous mutation (g.66496G>A) in exon 8 of the BMPR1B gene, without any amino acid change. Sequencing of the BMP15 gene revealed a deletion of 3 bp (g.656_658delTTC) in exon 1, leading to an amino acid deletion (p.Leu19del). Four single nucleotide polymorphisms (G1:g.2118G>A, G2:g.3451T>C, G3:g.3457A>G and G4:g.3701G>A), were detected in exons 1 and 2 of the GDF9 gene, two of which caused amino acid substitutions (G1: p.87Arg>His and G4: p.241Glu>Lys). These amino acid alterations are proposed to have a benign impact on structure and function of the GDF9 polypeptide sequence.

**Conclusion:**

Three major prolificacy genes (BMPR1B, BMP15 and GDF9) were polymorphic in Lori-Bakhtiari sheep, although none of the major causative mutation was detected in this sheep type. Further studies using high throughput methods such as genome-wide association study (GWAS) and evaluation of other candidate genes are necessary in the future.

## Introduction

Reproductive traits such as ovulation rate and litter size are genetically influenced by several minor genes as well as some major genes, called fecundity (Fec) genes (1). Three major genes including bone morphogenetic protein receptor 1B (*BMPR1B*) or FecB, bone morphogenetic protein 15 (*BMP15*) or *FecX* and growth differentiation factor 9 (*GDF9*) or FecG, belong to the transforming growth factor-beta (TGF-â) superfamily, located on ovine chromosomes 6, X and 5, respectively, and they affect the prolificacy (2).

Different causative mutations in the exon 8 of *BMPR1B (FecB),* both exons 1 and 2 of the *BMP15 (FecX^G^, FecX^B^, FecX^I^, FecX^H^, FecX^L^, FecX^R^, FecX^O^, FecX^Gr^)* and *GDF9* (FecGH, FecGT, FecGE, FecGNW) with major effects on ovulation rate and litter size have thus far been distinguished in various sheep breeds around the world (3). In this regard, the importance of the *BMP* system as an intraovarian regulator of follicular growth and maturation has been described (4, 5).

Lori-Bakhtiari sheep is an important heavyweight indigenous breeds, mainly raised in a wide range of Zagrous Mountains in southwestern part of Iran, with a current census over 1.7 million heads. This breed has the largest fat-tail size among all Iranian sheep breeds. Lori-Bakhtiari sheep is well known for providing a major source of meat with an average litter size of 1.17 ± 0.38 at birth and a conception rate of more than 90 percent (6). Considering that increasing the reproductive ability of the Lori-Bakhtiari sheep has always been an important breeding goal, genetic strategies have currently focused on reproduction traits to improve the profitability of sheep operations.

The aim of this study was to identify the possible presence of known main mutations in *BMPR1B, BMP15* and *GDF9* genes affecting reproductive performance in Lori-Bakhtiari sheep.

## Materials and Methods

### Experimental animals and DNA isolation

In the present experimental study, a total of 10 prolific Lori-Bakhtiari ewes with at least two twinning records in the first four parities were selected from different half-sib families at Sholi Sheep Breeding Station, Charmahal and Bakhtiari province, Iran. From the prolificacy point of view, the average of twinning rate in the studied population was about 18%. Blood samples were collected from jugular vein (5 ml per ewe) by venoject tubes contained ethylene diamine tetra acetic acid (EDTA) and immediately transported to the laboratory with ice before DNA isolation. Genomic DNA was extracted from whole blood by the CinnaGen DNP kit (CinnaGen Co, Iran).

All protocols were adhered in accordance with the ethical standards of the National Research Council’s 2011 guideline for the care and use of animals, approved by the research Ethics Committees of University of Guilan (Guilan, Iran) and Bu-Ali Sina University (Hamedan, Iran).

### Polymerase chain reaction amplification and sequence analysis

Five pairs of primer (Table 1) were designed to amplify exon 8 of *BMPR1B* (Gene ID: 443454) as well as exons 1 and 2 of both *BMP15* (Gene ID: 100141303) and *GDF9* (Gene ID: 100217402) genes, using Primer3 software online.

**Table 1:** Primer sequences


Gene	Region	Primer sequence (5ʹ-3ʹ)	Product size (bp)

*BMPR1B*	Exon 8	F: CCAGAGGACGATAGCAAAGCAA	190
		R: CAAGATGTTTTCATGCCTCATC	
*BMP15*	Exon 1	F: AAGCGTTATCCTTTGGGCTT	380
		R: CTGAGAGGCCTTGCTACACT	
	Exon 2	F: CGCTTTGCTCTTGTTCCCTC	906
		R: TAGCTGCACCTTTGCCGTC	
*GDF9*	Exon 1	F: GAAGACTGGTATGGGGAAATG	462
		R: CCAATCTGCTCCTACACACCT	
	Exon 2	F: TGGCATTACTGTTGGATTGTTT	1019
		R: GGTTTTACTTGACAGGAGTCTG	


Polymerase chain reaction (PCR) amplification protocols were similar in designated regions and they were carried out in a 50 μl volume, consisting of 25 μl Taq DNA Polymerase Master Mix 2X (CinnaGen, Iran), 10 pM of each primer (OD:2), 50-100 ng of DNA template and distilled water. PCR reactions were run in Applied Biosystems thermal cycler (Life technologies, USA) under the following thermal condition: Initial denaturation at 95°C for 5 minutes, followed by 35 cycles consisting of denaturation at 95°C for 1 minute, annealing at 60°C for 45 seconds, extension at 72°C for 1 minute and a final extension at 72°C for 5 minutes.

**Fig.1:**
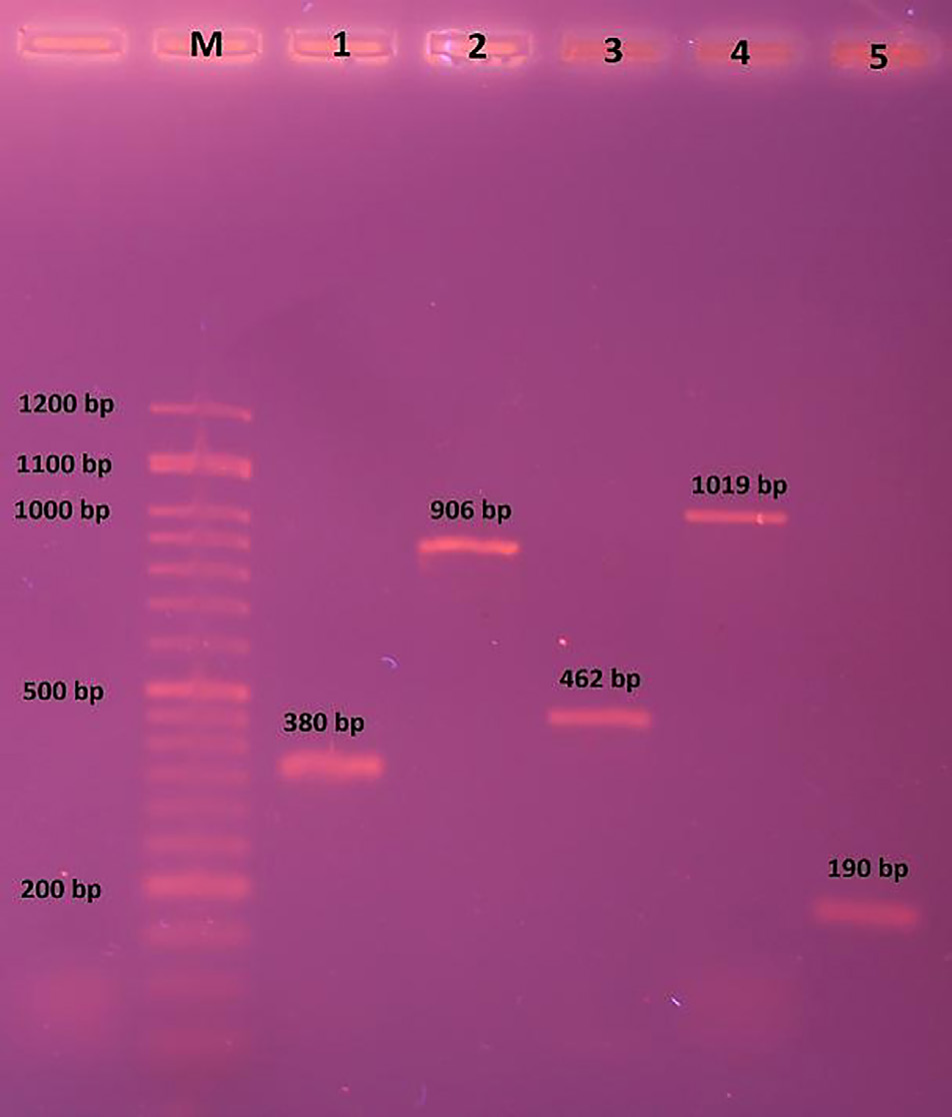
Polymerase chain reaction (PCR) products of *BMPR1B, BMP15* and *GDF9* genes bands. M; DNA molecular weight marker (Orange Ruler 50 bp DNA Ladder, CinnaGen, Iran), 1; 380 bp fragment of *BMP15* exon 1, 2; 906 bp fragment of *BMP15* exon 2, 3; 462 bp fragment of *GDF9* exon 1, 4; 1019 bp fragment of *GDF9* exon 2, and 5; 190 bp fragment of *BMPR1B* exon 8.

Individual fragments were distinguished by electrophoresis of PCR products in 2% agarose gel (CinnaGen, Iran). The gels were stained with ethidium bromide and photographed under UV light (BTS-20.M model, UVItec Ltd, UK) (Fig .1).

All 50 samples (five fragments from each animal) were submitted to DNA sequencing. The purified PCR products were sequenced on both strands by Bioneer Co., Korea. The identified single nucleotide polymorphisms (SNPs) were compared to the referring sequences at NCBI database using BLAST (7). Ultimately, any potential effect of the identified mutations, in terms of the structure and function of codified polypeptides, was predicted using PolyPhen-2 online software tool (8).

## Results

### Sequence analysis of the *BMPR1B* gene exon 8

**Fig.2:**
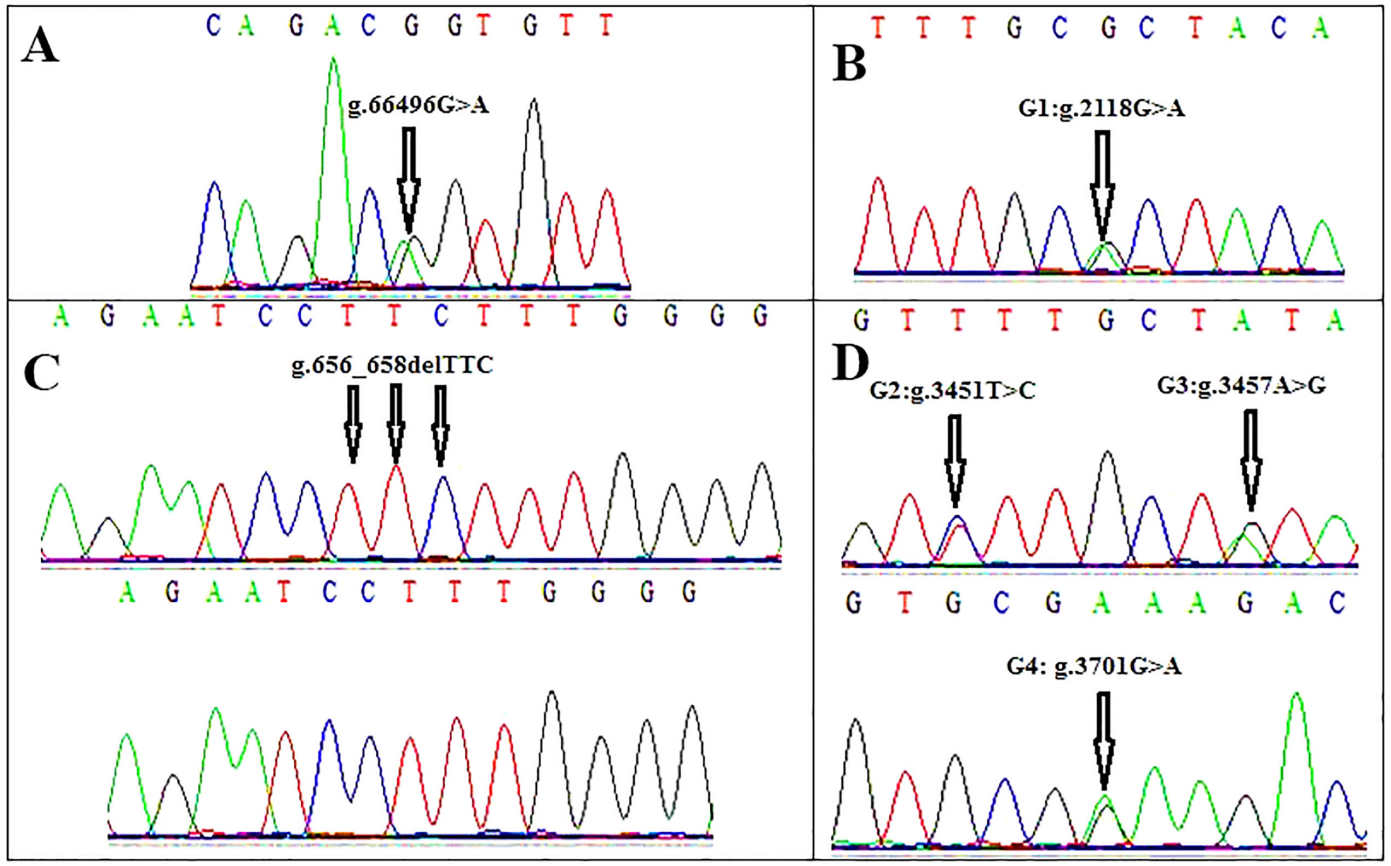
Sequencing chromatograms of the detected mutations of major prolificacy genes in Lori-Bakhtiari sheep. A. The identified transition (g.66496G>A) in *BMPR1B* exon 8, B. The identified 3bp deletion (g.656_658delTTC) in *BMP15* gene exon 1, C. The identified polymorphism in exon 1 of the *GDF9* gene (G1:g.2118G>A), and D. The identified polymorphisms in exon 2 of the *GDF9* gene (G2:g.3451T>C, G3:g.3457A>G and G4: g.3701G>A). Positions of the mutations are based on the full sequences of *BMPR1B, BMP15* and *GDF9* genes (Gene IDs; 443454, 100141303 and 100217402, respectively).

The sequence of exon 8 for *BMPR1B* gene did not show any FecB mutation corresponding with the increase of litter size and ovulation rate (9). However, the sequencing showed a novel synonymous mutation in the location of g.66496G>A (Fig .2). 

**Table 2:** Identified mutations of the major prolificacy genes in Lori-Bakhtiari sheep


Gene	Variation	Amino acid substitution	Frequency	Type of mutation

*BMPR1B* exon 8	g.66496G>A	-	2 out of 10	Synonymous
*BMP15* exon 1	g.656_658delTTC	p.Leu19del	1 out of 10	Non-synonymous
*GDF9* exon 1	g.2118G>A	p.87Arg>His	2 out of 10	Non-synonymous
*GDF9* exon 2	g.3451T>C	-	2 out of 10	Synonymous
	g.3457A>G	-	2 out of 10	Synonymous
	g.3701G>A	p.241Glu>Lys	2 out of 10	Non-synonymous


### Sequence analysis of *BMP15* gene exons 1 and 2

The sequence of exon 1 of *BMP15* showed a 3 bp nucleotide deletion (g.656_658delTTC) just in 1 out of 10 sheep samples (Fig .2). This mutation, leading to an amino acid deletion (p.Leu19de), has been described in Cambridge and Belclare sheep for the first time (10). So that nine sheep had two leucine codons and one showed this mutation with only one codon at this position (Table 2). No polymorphism was found in exon 2 of the *BMP15* gene.

### Sequence analysis of *GDF9* gene exons 1 and 2

**Fig.3:**
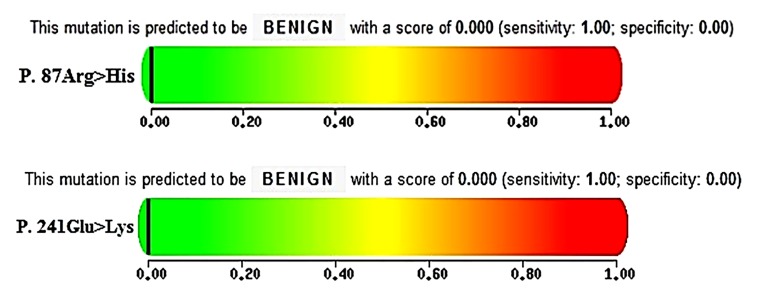
Prediction of the amino acid substitutions impact (G1: P.87Arg>His and G4: P.241Glu>Lys) on structure and function of the *GDF9* codified polypeptide.

Four single nucleotide polymorphisms (g.2118G>A, g.3451T>C, g.3457A>G and g.3701G>A), respectively known as G1-G4, were detected in the exons 1 and 2 of *GDF9* gene (Fig .2). Two of these mutations deduced amino acid changes, including G1: p.87Arg>His and G4: p.241Glu>Lys (Table 2). Potential effect(s) of the identified mutations on the structure and function of codified polypeptides were not significant, proposing no phenotypic effect (Fig .3).

## Discussion

Considering the hotspot regions involved in prolificacy of sheep, selection of a limited number of high prolific ewes and sequencing of their *BMPR1B* exon 8, in addition to the both exons 1 and 2 of *BMP15* and *GDF9* genes could be contemplated as an ideal approach to find related causative mutations. This approach would be less expensive than other methods and may lead to find new causative mutations in the indicated fragments. If this approach does not detect any important mutation, genome-wide association study (GWAS) implications would be a more appropriated method to determine causative mutations or candidate genes affecting prolificacy in the other parts of genome.

Evidences show that Booroola Merino ewes have high ovulation rate and litter size, due to the effects of a missense mutation (FecB) in the exon 8 of *BMPR1B* gene, located on chromosome 8 (9). This mutation has also been reported in Indian Garole and Kendrapada sheep breeds (11) as well as Chinese sheep breeds, Small Tail Han and Hu (12). The FecB was also found in Kalehkoohi sheep, as an Iranian breed (13).

In the present study, analysis of the sequences for *BMPR1B* gene exon 8 did not show the FecB alteration, as a mutation coordinating with increase of litter size and ovulation rate in the sheep. However, this analysis showed a new transition of g.66496G>A, compared to sequences reported for Garole (GenBank: GQ863576.1) and Booroola merino sheep, which is very close to the position of FecB mutation. This mutation causes a synonymous substitution which does not alter amino acid sequences. Western blot and qRT-PCR techniques could in future be used to verify possible effects of this SNP on translation and transcription aspects respectively. Moreover, linkage disequilibrium of this SNP with variants in other loci should be considered and investigated (14).

Eight different causative mutations in ovine *BMP15* gene with major effects on ovulation rate and litter size have previously been reported in literature (3). Hence, the *BMP15* gene could be considered as the most polymorphic locus among the major genes affecting prolificacy in sheep. As a case, evidences showed that a nonsense mutation, due to deletion of 17 bp nucleotides, altered the amino acid sequence and introduced a premature stop codon in this protein (15).

In the present study, sequencing of the exon 1 of *BMP15* gene showed 3 bp nucleotides deletion (g.656_658delTTC) in one sample, whereby nine sheep had two leucine codons at this position while this sheep showed heterozygote mutation. This mutation has been reported for the first time in Cambridge and Belclare sheep, without any determined significant effect on prolificacy (10). However, because of low frequency of this deletion and missing the infertile or singleton-bearing ewes, investigation of this aberration was not feasible in the present study. Further investigations demonstrated no polymorphism in the exon 2 of *BMP15* gene.

Hanrahan et al. (10) reported eight point mutations (i.e. G1-G8) in the *GDF9* gene of Cambridge and Belclare sheep, five of which deduced amino acid changes (G1: p.87Arg>His; G4: p.241Glu>Lys; G6: p.332Val>Ile; G7: p.371Val>Met and G8: p.395Ser>Phe), while only one of them (G8; also known as FecGH) had additive effects on prolificacy. The first mutation (G1: g.2118G>A) was also reported to associate with an increased ovulation rate and litter size in some sheep breeds (16, 17). In the present study, the mutations of G1 (g.2118G>A), G2 (g.3451T>C), G3 (g.3457A>G) and G4 (g.3701G>A) were identified in exons 1 and 2 of the *GDF9* gene. With exception for G1 mutation, which had an additive effect (16, 17), other mutations (G2, G3 and G4) did not carry any significant effect on litter size in some sheep breeds (18). The impacts of amino acid substitutions for two non-synonymous mutations (G1: p.87Arg>His and G4: p.241Glu>Lys) were also benign with scores of 0.00. In fact, the phenotypic expression of an allele, to some extent, depends on other alleles, mainly multiple interacting mutations, and thus, a phenotypic effect of an allele may be observed in one breed while being absent in the other (3, 19). For instance, a GWAS analysis showed a missense mutation in the *GDF9* (FecG^NW^) with strong association with litter size in Norwegian white sheep (20). This mutation, known as G7, has also been identified in Belclare and Cambridge sheep as well as G1, but without any phenotypic effects (10). Hence, more studies are still needed to determine the precise effects of this kind of mutations and epistatic effects evaluation.

Regarding the absence of known main causative mutations of *BMPR1B, BMP15* and *GDF9* genes in the studied population, high prolificacy could be attributed to the other genetic factors not studied here. Thus, seeking for other related major genes, such as a regulatory mutation in intron 7 of B4GALNT2 (Fec^L^) largely affecting the respective gene expression in prolific ewes and most likely taking responsibility for high prolificacy in Lacaune sheep (1), could be considered as an interesting subject for similar studies in the future. However, regarding the polygenic control of reproductive traits, GWAS and evaluation of linkage disequilibrium with other mutations or transcriptome analysis are also necessary to understand the precise underlying pathway(s) of prolificacy in Lori-Bakhtiari sheep.

## Conclusion

We can state that major prolificacy genes (i.e. *BMPR1B, BMP15* and *GDF9*) were polymorphic in the studied population of Lori-Bakhtiari sheep, but none of the previously identified mutations contributing to prolificacy was detected in the studies genes. Moreover, no clear statistical association was determined among the observed polymorphisms and prolificacy. More studies on other candidate genes or use of high throughput methods such as GWAS are necessary in the future studies.
